# Glomerular Complement Factor H–Related Protein 5 (FHR5) Is Highly Prevalent in C3 Glomerulopathy and Associated With Renal Impairment

**DOI:** 10.1016/j.ekir.2019.06.008

**Published:** 2019-06-19

**Authors:** Nicholas R. Medjeral-Thomas, Hilary Moffitt, Hannah J. Lomax-Browne, Nicholas Constantinou, Tom Cairns, H. Terence Cook, Matthew C. Pickering

**Affiliations:** 1 Centre for Inflammatory Disease, Division of Immunology and Inflammation, Department of Medicine, Imperial College London, UK; 2 Renal and Transplant Centre, Imperial College Healthcare NHS Trust, UK

**Keywords:** complement, C3 glomerulopathy, glomerulonephritis

## Abstract

**Introduction:**

Therapeutic agents that target complement are increasingly available for glomerular diseases. However, the mechanisms linking glomerular complement deposition with inflammation and damage are incompletely understood. Complement factor H–related protein 5 (FHR5) interacts with complement C3 and is considered to promote activation. Circulating and glomerular FHR5 associates with IgA nephropathy and abnormal FHR5 associates with familial C3 glomerulopathy (C3G). We characterized glomerular FHR5 staining in C3G and assessed its relationships with histological features of glomerular injury and clinical outcome.

**Methods:**

We developed FHR5 staining protocols for formalin-fixed paraffin-embedded (FFPE) renal tissue and applied them to surplus biopsy sections from a C3G cohort.

**Results:**

Glomerular FHR5 was highly prevalent in native and transplant C3G and correlated with glomerular C3 and C5b-9 staining. Glomerular FHR5 staining correlated negatively with estimated glomerular filtration rate (eGFR) (*P* = 0.04, difference of medians 19.7 ml/min per 1.73 m^2^; 95% confidence interval [CI] 1.1–43.0) and positively with a membranoproliferative glomerulonephritis pattern at diagnostic biopsy (odds ratio 18; 95% CI 1.6–201; *P* = 0.049). Glomerular FHR5 staining intensity positively correlated with glomerular complement C3b/iC3b/C3c (Pearson’s correlation coefficient [*R*] = 0.59; *P* = 0.0008), C3dg (*R* = 0.47; *P* = 0.02) and C5b9 (*R* = 0.44, *P* = 0.02).

**Conclusions:**

Glomerular FHR5 is highly prevalent in C3G, interacts with glomerular C3, and is associated with markers of disease severity. Glomerular FHR5 likely exacerbates complement-mediated glomerular damage in C3G and its interaction with glomerular complement might be exploited to target complement therapeutic agents.

## Graphical abstract

**Figure:**
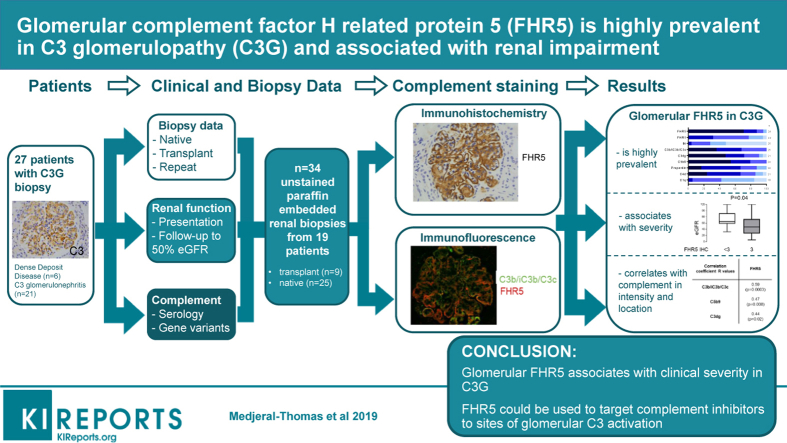

See Commentary on Page 1359

Complement dysregulation is associated with renal disease, which includes atypical hemolytic uremic syndrome and C3G.^1^, ^2^ Drugs that target specific complement proteins and pathways have emerged as therapeutic options for complement-associated renal diseases.^3^ Complement C5 inhibition is an effective therapy for atypical hemolytic uremic syndrome, a condition characterized by complement-mediated glomerular thrombosis.^2^ However, the mechanisms through which complement dysregulation contribute to glomerular inflammation are incompletely understood. This limits our ability to select patients for specific complement inhibitors and monitor disease activity. For example, the response of C3G to complement C5 inhibition is heterogeneous.^2^

C3G is associated with significant morbidity. Up to 50% of C3G patients progress to end-stage renal disease within 5 years of diagnosis,^4^, ^5^ and recurrent C3G frequently causes transplant dysfunction.^6^, ^7^ Abnormal regulation of complement alternative pathway activation is seen in C3G.^8^, ^9^, ^10^ This can be the result of genetic and acquired factors. However, in many cases the cause is unknown. The key negative regulator of C3 activation through the alternative pathway is complement factor H (fH). The fH protein family includes the 5 fH-related proteins (FHR1–5), named because of their structural similarity to fH.^11^ Because they lack the complement-regulatory domains of fH but retain C3-binding domains,^12^ FHR proteins are currently thought to act as positive regulators that promote complement activity. A heterozygous mutation in FHR5 is associated with a familial form of C3G, known as CFHR5 nephropathy.^13^ A number of other FHR protein variants have been identified in association with cases of C3G.^14^, ^15^, ^16^, ^17^, ^18^

C3G includes dense deposit disease (DDD) and C3 glomerulonephritis. The range of histopathology features encompassed by a diagnosis of C3G has uncertain relevance to outcome. In some studies, DDD has been associated with a higher incidence of progression to end-stage renal disease, whereas in a recent cohort no difference between these subsets was evident.^5^, ^6^ It is also unclear whether or not glomerular deposition of specific complement proteins influences C3G outcome. Complement activation results in the cleavage of C3 to C3b, which can covalently attach to glomerular tissue. The C3b can contribute to further C3b generation through a positive feed-back loop or it can be cleaved sequentially to iC3b and then to C3dg, releasing C3c. Glomerular C3c is cleared within 24 hours after stopping complement activation in experimental glomerulonephritis models,^19^ whereas C3dg persists. Glomerular FHR5 has been documented in a similar staining distribution to glomerular C3 in a range of glomerulopathies, including C3G and IgA nephropathy.^20^ FHR5 binds C3b, iC3b, and C3dg and demonstrates stronger binding than fH for iC3b and C3dg.^21^ In addition to the associations between FHR5 variants and familial C3G,^13^, ^15^, ^22^ evidence that glomerular FHR5 might increase glomerular injury includes *in vitro* studies demonstrating FHR5 can (i) bind extracellular matrix components and support assembly of the alternative pathway C3 convertase formation, thereby promoting further C3b generation, and (ii) antagonize the ability of fH to limit amplification of C3b (a process termed fH deregulation).^21^, ^23^, ^24^ Furthermore, both circulating FHR5 levels^25^, ^26^ and the intensity of glomerular FHR5 deposition^27^ associate with IgA nephropathy severity.

In this study, we characterized the spectrum of glomerular FHR5 deposition in C3G in native and transplant kidneys, its relationships with other complement components, and, where possible, its change over time in the same patient. We show that glomerular FHR5 is highly prevalent in both native and transplant kidney C3G, colocalizes with C3b/iC3b/C3c and C3dg *in vivo,* and associates with disease severity.

## Methods

### Patient Cohort

We reviewed the diagnosis of all kidney biopsies performed at Imperial College Healthcare NHS Trust over a 20-year period. This totaled 6592 native and 2577 transplant biopsies. We included all patients with a biopsy diagnosis of C3G. After review of each case, we excluded patients with thrombotic microangiography, immune-complex dependent disease, or membranoproliferative glomerulonephritis (MPGN) type 1. We derived clinical and pathology data from medical records. Ethical approval was through the Natural History Study of C3 Glomerulopathy: Discovery of Histological Predictors of Outcome study (UK National Research Ethics Service reference 16/WM/0497) and the Imperial College Healthcare Tissue Bank. The Imperial College Healthcare Tissue Bank is supported by the National Institute for Health Research Biomedical Research Centre based at Imperial College Healthcare NHS Trust and Imperial College London. The Imperial College Healthcare Tissue Bank is approved by the National Research Ethics Service to release human material for research (12/WA/0196). Outcome parameters were 50% of eGFR from presentation and end-stage renal disease (defined as commencing dialysis or receiving a renal transplant). Patients with less than 12 months of follow-up were excluded from outcome analyses. eGFR was calculated using the Chronic Kidney Disease Epidemiology Collaboration equation^28^ for adults and the Schwartz formula^29^ for children and corrected to a maximum of 120 ml/min per 1.73 m^2^. Nephrotic syndrome was defined as a combination of serum albumin of less than 32 g/l, edema, and urine protein:creatinine ratio of at least 250 mg/mmol or 24-hour urine proteinuria of at least 3 g. We defined crescentic C3G as the presence of cellular or fibrocellular crescents in at least 50% of glomeruli.

### Immunohistochemistry

FFPE renal biopsy sections were examined for the following proteins: FHR5, FHR1, fH, C3b/iC3b/C3c, C3dg, C5b9, properdin, C4d, C1q, and CD68. Primary antibodies were polyclonal rabbit anti-C3c (#A0062; Dako, Glostrup, Denmark); polyclonal rabbit anti-C3d (#136916; Abcam, Cambridge, UK) that recognizes C3dg; polyclonal rabbit anti-properdin (#2097; Biorbyt, Cambridge, UK); monoclonal mouse anti-C5b9 (#M0777; Dako); polyclonal rabbit anti-C4d (#107-01; BD Biotech, Franklin Lakes, NJ); monoclonal rabbit anti-C1q (#A0136; Dako); monoclonal mouse anti-factor H, OX-24 (#118820; Abcam); monoclonal mouse anti-FHR1 (#3078-M01; Abnova, Taipei, Taiwan); polyclonal rabbit anti-FHR5 (#81494-D01P; Abnova); and monoclonal mouse anti-CD68 (#M0876; Dako). Variation in glomerular FHR5 staining between nonsclerosed glomeruli from the same biopsy was minimal with bacterial enzyme antigen retrieval. FHR5 staining was eliminated after pre-incubation of the primary antibody with purified FHR5 (#3845-F5; R&D Systems, Minneapolis, MN; Supplementary Figure S1). The anti-FHR5 antibody showed minimal binding to plate-bound purified human C3c (#A116; Comptech, Tyler, TX), iC3b (#A115; Comptech), and C3d (#A117; Comptech) in the absence of purified FHR5 (Supplementary Figure 2A). The anti-C3c antibody (#A0062; Dako) recognized plate-bound purified human C3c and iC3b, so we refer to this antibody as anti-C3b/iC3b/C3c. This anti-C3b/iC3b/C3c antibody and the anti-C3d antibody (#136916; Abcam) recognized plate-bound human C3 fragments irrespective of the presence of FHR5 (Supplementary Figure 2B and C). For pre-incubation experiments, we used recombinant full-length human FHR5 (#3845-F5; R&D Systems) and heparin sodium (#H3393; Sigma-Aldrich, St. Louis, MO) at 30 times the antibody concentration.

Two-micron thickness sections from FFPE renal tissue were fixed on Polysine slides, deparaffinized, and hydrated (xylene for 10 minutes followed by an ethanol gradient), washed in distilled water, and subjected to antigen retrieval. For C3b/iC3b/C3c, C5b9, and FHR5 (Supplementary Figure 1): bacterial proteinase XXIV (#P8038; Sigma-Aldrich) at 37 °C for 30 minutes. For C3dg, C4d, fH, and C1q: sodium citrate tribasic buffer pH 6 at 95 °C for 30 minutes. For properdin, FHR1, and CD68: Tris-EDTA buffer at pH 9 and heating in a pressure cooker for 30 minutes. Sections were blocked for 60 minutes at room temperature with hydrogen peroxidase (EnVision, #K4007 or #K4011; Dako) followed by 30% normal goat serum. Primary antibody was applied overnight at 4 °C followed by application of secondary antibodies for 60 minutes at room temperature: horseradish peroxidase–conjugated goat anti-mouse (EnVision+System-HRP, #K4007; Dako) or anti-rabbit (EnVision+System-HRP, #K4011; Dako) IgG. 3ʹ-Diaminobenzidine substrate (EnVision+System-HRP, #K4007 or #K4011; Dako) was applied for up to 15 minutes and sections counterstained with filtered hematoxylin, washed and dehydrated and cleared in xylene for 10 minutes before mounting using xylene-based media (Pertex, #SEA-0104–00A; CellPath, Newtown, UK).

Antigen staining intensities were graded as previously described.^27^ Because nonspecific staining was seen in sclerosed glomeruli in previous^20^ and our studies, only nonsclerosed glomeruli were included in statistical analyses. We tested for correlations between staining intensities and clinicopathologic markers of disease severity: MPGN light microscopy pattern; urine protein:creatinine ratio at biopsy; eGFR at biopsy; loss of 50% eGFR during follow-up; change in eGFR during follow-up; end-stage renal disease during follow-up.

### Immunofluorescence

Antibody combinations for double antigen immunofluorescence (IF) staining were as follows: C3c and C3dg: fluorescein isothiocyanate–conjugated sheep polyclonal anti-human C3c antibody (#PA1–36179; ThermoFisher, Waltham, MA) and rabbit polyclonal anti-C3d (#136916; Abcam) with Alexa Fluor AF555-conjugated goat anti-rabbit IgG (#A-21429; ThermoFisher). C3c and FHR5: fluorescein isothiocyanate–conjugated sheep polyclonal anti-human C3c antibody (#PA1–36179; ThermoFisher) and mouse monoclonal anti-human FHR5 antibody (#81494-B01P; Abnova) with AF555-conjugated goat anti-mouse IgG (#150118; Abcam). C3dg and FHR5: rabbit polyclonal anti-C3d (#136916; Abcam) with AF555-conjugated goat anti-rabbit IgG (#A-21429; ThermoFisher) and mouse monoclonal anti-human FHR5 antibody (#81494-B01P; Abnova) with AF488-conjugated goat anti-mouse IgG (#A-11029; ThermoFisher). The anti-C3c antibody used for IF staining recognized plate-bound C3c and iC3b irrespective of the presence of purified human FHR5 and will also be referred to as anti-C3b/iC3b/C3c (Supplementary Figure 2D). The anti-C3d antibody used for immunohistochemistry and IF showed relatively selective binding to plate-immobilized C3d (Supplementary Figure 2C). The anti-C3b/iC3b/C3c used for IF staining showed relatively selective binding to plate-immobilized C3c and iC3b (Supplementary Figure 2D).

Staining was performed using 2- to 4μm-thick FFPE sections fixed on Polysine slides, deparaffinized (xylene for 10 minutes followed by an ethanol gradient), washed in distilled water, and subjected to antigen retrieval: immersion in sodium citrate tribasic buffer pH 6 at 95 °C for 30 minutes and incubation with HistoReveal (#103720; Abcam) for 5 minutes at room temperature. Slides were blocked with 2% bovine serum albumin in phosphate-buffered saline for 60 minutes at room temperature and the first antibody applied overnight at 4 °C. Sections were blocked again with 2% bovine serum albumin at 4 °C for 8 hours followed by incubation of the second antibody overnight at 4 °C. Sections were sequentially incubated with the appropriate secondary Alexa Fluor antibodies, nuclear stain (4ʹ,6-Diamidino-2-Phenylindole, Dilactate, #D3571; ThermoScientific, Waltham, MA), 0.1% Sudan Black B (#199664; Sigma, St. Louis, MO) in 70% ethanol to reduce autofluorescence and mounted using Mowial 4–88 (#475904; Calbiochem, San Diego, CA).

We used ImageJ software (National Institutes of Health, Bethesda, MD) and the COLOC2 plug-in^30^ to calculate antigen location correlations. This analyzed the antigen colocalization of each pixel within the image region of interest. We ensured IF images were captured with identical microscope settings, images were of identical size, and the brightness threshold at which monochromatic (red or green) fluorescence was counted as positive deposition was standardized. The glomerulus from each section was selected as the region of interest for analysis. Pearson’s correlation coefficients were recorded for each antigen combination in all glomeruli. For each antibody combination, this provided a number of antibody location correlation coefficients, which quantified how closely 2 antibodies colocalized on the same tissue section. We wanted to compare how closely each pair of antibodies colocalized. For example, we questioned whether the C3b/iC3b/C3c and C3d antibodies colocalized more or less closely than FHR5 and C3d. To do this, we calculated the median of the correlation coefficients for each antibody pair, and then compared these median values.

### Statistical Analysis

We used Pearson’s correlation coefficient to calculate IF antigen location correlations with ImageJ software.^30^ We used GraphPad (La Jolla, CA) Prism Version 6.00 for Windows for all other statistical analyses. Normally distributed continuous variables were compared using either unpaired *t*-test or (for multiple groups) 1-way analysis of variance. Continuous variables with skewed distribution were compared using either Mann-Whitney *U* test or (for multiple groups) Kruskal-Wallis test. We calculated CIs using the Hodges-Lehmann method and adjusted for multiple analyses with the 2-stage linear step-up procedure of Benjamini *et al.*^31^ Boxplots were used to display correlation data. We included repeat biopsies from the same patients because we were interested in the correlation of glomerular FHR5 with other complement proteins and eGFR at each biopsy point. The dependence of these outcomes on repeated measures is not known.

## Results

### C3G Cohort

We identified 27 individuals with biopsy-proven C3G (Tables 1^32^, ^33^ and 2,^13^). Two patients were lost to follow-up. A 50% loss of eGFR during follow-up occurred in 14 of 25 patients (56.0%). A MPGN light microscopy pattern at diagnostic biopsy associated with 50% loss of eGFR during follow (*P* = 0.02, *n* = 25, odds ratio for 50% loss of eGFR = 9.8 for MPGN at diagnosis compared with non-MPGN light microscopy patterns; 95% CI 1.5–47.1). Patients with 50% loss of eGFR during follow-up had significantly lower median eGFR at diagnostic biopsy (45.0 vs. 117.7 ml/min per 1.73 m^2^; *P* = 0.049, *n* = 25, difference of median eGFR −72.7 ml/min per 1.73 m^2^; 95% CI −0.2 to −76.6) but no significant difference in the amount of proteinuria at biopsy (Table 1). Surplus tissue was available from 34 renal biopsies from 19 patients. Nine of these were transplant biopsies from 5 patients. Four of the 19 patients (21.1%) with available biopsy tissue had a diagnosis of DDD.

**Table 1:** Clinical and histology features

Characteristic	Number	Percentage	Median (range)
**Biopsy at center**	27		
Age, yr			30.4 (9.4–73.8)
Male	14	51.9	
White	16	59.3	
eGFR (ml/min per 1.73 m^2^)			74 (5–120)^a^
Urine protein:creatinine ratio (mg/mmol)			263 (0–1653)
Serum albumin (g/l)			29 (11–43)
Hematuria (macro- and microscopic)	16	59.3	
Nephrotic syndrome	14	51.9	
Paraproteinemia (*n* = 19)	3	17.6	
**Diagnostic biopsy features**	27		
Glomeruli per diagnostic biopsy			17 (5–46)
Percentage sclerosed glomeruli			10% (0%–57%)
Dense deposit disease	6	22.2	
Light microscopy pattern:			
Mesangial proliferative	11	40.7	
Endocapillary proliferative	5	18.5	
MPGN	16	59.3^b^	
Other	2	7.4	
Cellular crescents	7	25.9	
Endocapillary hypercellularity	17	63.0	
Tubular atrophy:			
None	5	18.5	
1%–10%	9	33.3	
11%–25%	8	29.6	
26%–50%	5	18.5	
Detectable immunofluorescence			Mean intensity (range)
C3	27	100.0	2.4 (1–3)
C1q	9	33.3	0.4 (0–1)
IgA	1	3.7	0.04 (0–1)
IgG	4	14.8	0.1 (0–1)
IgM	13	48.1	0.5 (0–2)
**Treatment after diagnostic biopsy (*n* = 28)**	25		
ACEi/ARB	20	80.0	
Any immunosuppression (including corticosteroids)	16	64.0	
- Corticosteroids	14	56.0	
- Mycophenolate mofetil	9	36.0	
- Cyclophosphamide	7	28.0	
**Follow-up**	25		
Duration follow-up (mo)			77.9 (31–155.7)
Loss of 50% eGFR at follow-up	14	56.0	
Time diagnosis to 50% eGFR loss (mo)			12.9 (0.1–50.8)
ESRD	12	48.0	
Time diagnosis to ESRD (mo)			40.0 (1–69.2)
Transplant	9	36.0	
Transplant recurrence of C3G (*n* = 9)	9	100.0	
Time transplant to recurrence (*n* = 9) (mo)			4.9 (1–26.8)
Recurrence within 6 mo (*n* = 9)	4	44.4	
Recurrence within 18 mo (*n* = 9)	9	100	

ACEi, angiotensin-converting-enzyme inhibitor; ARB, angiotensin II receptor blocker; eGFR, estimated glomerular filtration rate; ESRD, end-stage renal disease; MPGN, membranoproliferative glomerulonephritis.

eGFR calculated with the Chronic Kidney Disease Epidemiology Collaboration equation^28^ for adults and the Schwartz formula^29^ for children and corrected to a maximum of 120 ml/min per 1.73 m^2^.

a Associates with 50% loss of eGFR at follow-up. Of the 25 patients with follow-up data available, patients with 50% loss of eGFR during follow-up had median eGFR at diagnostic biopsy of 45.0 versus 117.7 ml/min per 1.73 m^2^ for those without (*P* = 0.049, 95% confidence interval −0.2 to −76.6).

b Associates with 50% loss of eGFR at follow-up. Of the 25 patients with follow-up data available, 78.6% of patients with 50% loss of eGFR during follow-up had MPGN on biopsy versus 27.3% of those without 50% eGFR loss (*P* = 0.02; odds ratio = 9.8; 95% confidence interval 1.5–47.1).

**Table 2:** Complement analyses

Complement assessment at diagnostic biopsy	Number tested	Percentage of cohort	Number	Percentage	Median (range)
Serum C3, g/l (NR 0.7–1.7)	25	100			0.77 (0.10–1.91)
Low serum C3	25	100	11	44.0	
Serum C4, g/l (NR 0.16–0.54)	25	100			0.27 (0.14–0.4)
Low serum C4	25	100	1	4.0	
CH-50, %	22	88			81 (20–130)
CH-50 <50%	22	88	6	27.3	
AP-50 (%)	22	88			84.0 (20–115)
AP-50 <50%	22	88	9	40.9	
Anti-C1q antibody	10	40	4	40.0	
Serum factor H, mg/l	22	88			597 (286–800)
Serum factor I, mg/l	22	88			49 (26–76)
C3 nephritic factor	24	96	2	8.3	
Anti-fH antibody	22	88	1	4.5	
Complement gene variant^a^	12	48	4	33.3	

fH, complement factor H; NR, normal range.

a Heterozygous factor I mutation (*n* = 1, c.57719G>T, p.(Gly367STOP) variant of uncertain significance); heterozygous C3 mutation (*n* = 1, c.463A>C, p.(Lys155Gln) likely pathogenic C3 gene mutation); FHR5 nephropathy^13^ (*n* = 2, duplication of exons 2 to 3 of *CFHR5* gene in heterozygosity).

### Glomerular FHR5 is Highly Prevalent in C3G

**Figure 1:**
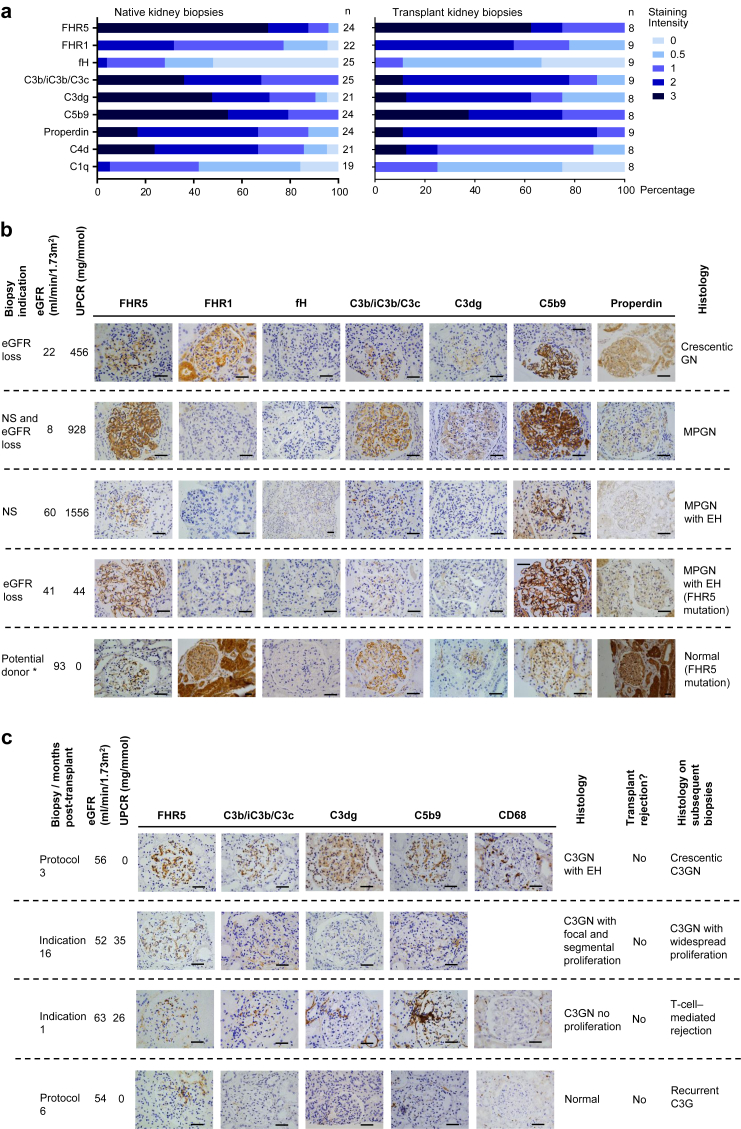
Glomerular FHR5 staining in C3 glomerulopathy. (a) Glomerular staining intensity in native and transplant biopsies. FHR5 was the most frequent protein at 3+ intensity and detected in all of the transplant biopsies. (b) Representative images of complement staining in the native kidney. For each set of images, the biopsy indication, the appearances of the glomeruli by light microscopy, and the estimated glomerular filtration rate (eGFR) and proteinuria at the time of biopsy are indicated. *The cases represented by the fourth and fifth rows of images are from the same family. (c) Representative images of complement staining in transplant kidneys. For each set of images, the biopsy indication (either protocol or due to clinical changes; i.e., indication biopsy), time posttransplant together with the eGFR, and proteinuria at the time of biopsy are indicated. The histology including the presence or absence of rejection and the histology of subsequent biopsies is also shown. C3G, C3 glomerulopathy; C3GN, C3 glomerulonephritis; EH, endocapillary hypercellularity; fH, factor H; FHR1, factor H–related protein 1; FHR5, factor H–related protein 5; FHR5 mutation, the mutation described in CFHR5 nephropathy^13^; MPGN, membranoproliferative glomerulonephritis; NS, nephrotic syndrome; UPCR, urinary protein:creatinine ratio. Original magnification ×400. Bars = 100 μm.

Using immunohistochemistry staining on FFPE renal biopsy sections,^27^ glomerular FHR5 was detected in all the transplant biopsies (*n* = 8 from 4 patients) and all but 1 of the native biopsies (23 of 24 biopsies from 15 patients). The sample sizes varied depending on the availability of surplus stored renal tissue. FHR5 was the most prevalent glomerular complement protein detected at 3+ intensity (Figure 1a–c). We did not detect significant differences in complement protein immunostaining when comparing cases of DDD with C3GN. We have previously reported the prevalence of glomerular FHR5 deposition in IgA nephropathy.^27^ We did not detect glomerular FHR5 in 3 thin basement membrane cases (Supplementary Figure S2).

We detected glomerular FHR5, C3, and C5b-9 in 2 cases of CFHR5 nephropathy, 1 with and 1 without glomerular inflammation and clinical signs of glomerulonephritis (Figure 1b). Although C5b-9 and FHR5 staining intensities were greater in the biopsy with glomerular inflammation, the detection of C5b-9 and FHR5 in a potential kidney donor with normal renal function but subclinical CFHR5 nephropathy shows that glomerular complement deposition can be detected in the absence of glomerular inflammation in CFHR5 nephropathy.

**Figure 2:**
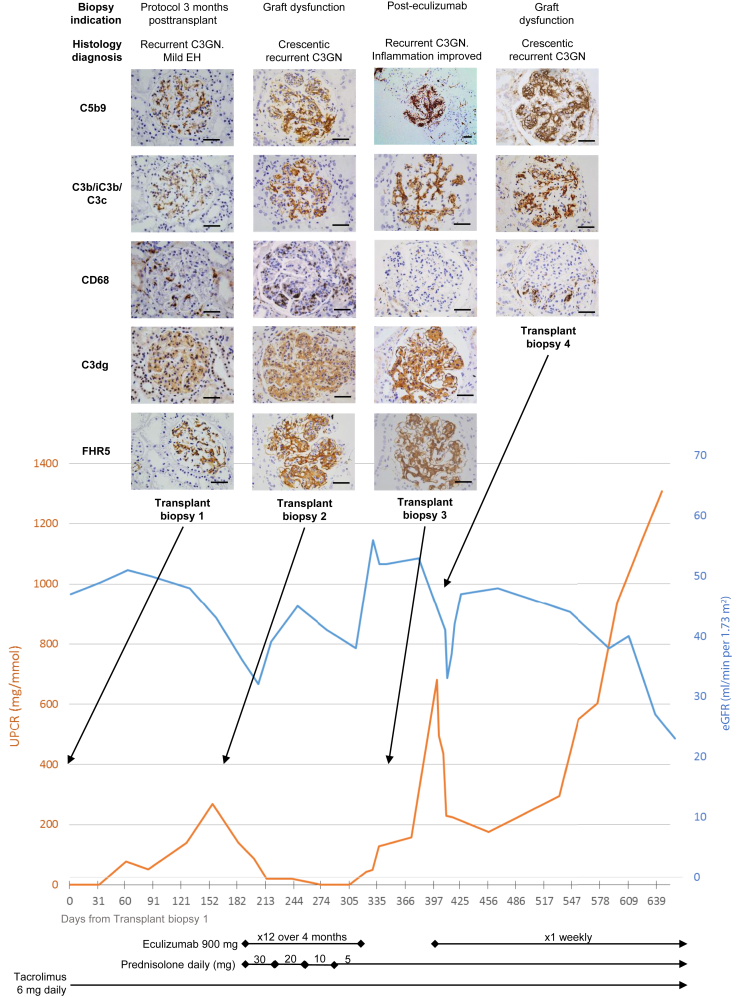
Sequential glomerular FHR5 staining in a single case of C3 glomerulopathy transplant recurrence. Images and information from the same biopsy are organized in columns. A protocol surveillance biopsy performed 3 months after transplantation (Transplant biopsy 1) showed recurrent C3 glomerulonephritis (C3GN) with mild endocapillary hypercellularity (EH). Glomerular factor H–related protein 5 (FHR5), C5b9, C3b/iC3b/C3c, and C3dg were detected. The estimated glomerular filtration rate (eGFR) was stable and there was no significant proteinuria. Approximately 6 months posttransplant, the patient developed proteinuria and a fall in eGFR. Biopsy showed crescentic C3GN and increased glomerular CD68-positive cells (Transplant biopsy 2). Glomerular FHR5, C5b9, C3b/iC3b/C3c, and C3dg were detected but at increased staining intensity compared with the first transplant biopsy. The eGFR and proteinuria improved with eculizumab and prednisolone treatment. After 4 months of eculizumab treatment, renal biopsy (Transplant biopsy 3) showed resolution of glomerular CD68 staining, but glomerular staining for FHR5, C5b9, C3b/iC3b/C3c, and C3dg remained unchanged. After a further 3 months, proteinuria increased and biopsy showed crescentic C3GN and the recurrence of glomerular CD68-positive cells (Transplant biopsy 4). Proteinuria improved with re-introduction of eculizumab. UPCR, urine protein:creatinine ratio. Original magnification ×200 or ×400. Bars = 100 μm.

Two of the renal transplant biopsies were part of rejection surveillance programs (“protocol biopsy”), thereby providing an opportunity to characterize subclinical C3G (Figure 1c). None of the C3G patients with renal transplant had a diagnosis of antibody-mediated rejection. In 1 protocol biopsy performed 6 months posttransplant, the glomeruli were normal by light microscopy and glomerular C3, C5b9, and CD68 staining were negative. However, glomerular FHR5 staining was detectable. Nine months later, the patient developed biopsy-proven C3G recurrence (surplus biopsy tissue unavailable to study). The other protocol biopsy was from an individual who had a 3 further transplant biopsies and treatment with eculizumab. The initial protocol biopsy revealed subclinical C3G recurrence with glomerular endocapillary hypercellularity and glomerular staining for C3b/iC3b/C3c, C3dg, C5b-9, FHR5, and CD68 (Figure 2). A biopsy performed due to proteinuria and fall in eGFR 6 months later showed crescentic C3G and increased glomerular C3b/iC3b/C3c, C3dg, C5b-9, FHR5, and CD68 staining (Figure 2, transplant biopsy 2). A biopsy 4 months after starting eculizumab treatment showed reduced endocapillary hypercellularity and reduced glomerular CD68 infiltration (Figure 2, transplant biopsy 3). Proteinuria and eGFR had improved but glomerular FHR5, C3b/iC3b/C3c, C3dg, and C5b-9 staining were unchanged. Three months after stopping eculizumab, graft function deteriorated again, and a fourth transplant biopsy showed recurrent crescentic C3GN. Rapid clinical improvement was seen after reintroducing eculizumab treatment, but subsequently proteinuria increased despite ongoing eculizumab therapy.

### Glomerular FHR5 Colocalizes With Glomerular C3 in C3G

**Figure 3:**
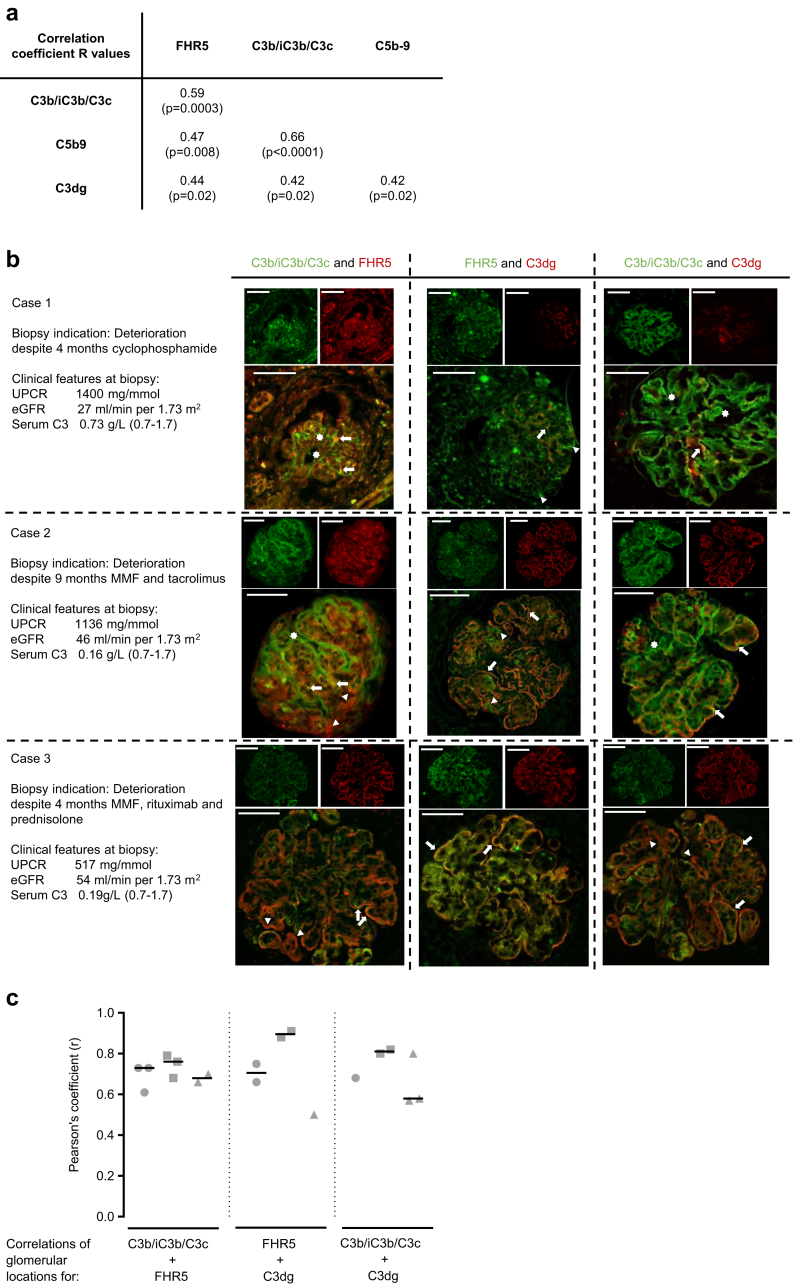
Glomerular factor H–related protein 5 (FHR5) colocalizes with C3 in C3 glomerulopathy. (a) Glomerular FHR5 staining intensity positively correlated with the staining intensity of C3b/iC3b/C3c, C3dg, and C5b9. Both glomerular C3b/iC3b/C3c and C3dg positively correlated with glomerular C5b9. *R* values are calculated from Pearson’s correlation coefficient and *P* values are adjusted for multiple comparisons. (b) Representative images of combined immunofluorescence staining in three C3G cases for glomerular FHR5 with either C3b/iC3b/C3c or C3dg, and C3b/iC3b/C3c with C3dg. Renal biopsies in all 3 cases showed C3-dominant membranoproliferative glomerulonephritis, and the biopsy indications together with the urine protein:creatinine ratio (UPCR), estimated glomerular filtration rate (eGFR), and serum C3 levels at the time of biopsy are listed. The staining patterns for C3b/iC3b/C3c and C3dg (right-hand column of images) showed areas of colocalization (arrows), areas of C3b/iC3b/C3c alone (stars), and areas of C3dg alone (triangles). The staining pattern for C3b/iC3b/C3c and FHR5 (left-hand column of images) showed areas of colocalization (arrows), areas of C3b/iC3b/C3c alone (stars), and areas of FHR5 alone (triangles). The staining pattern for C3dg and FHR5 (middle column of images) showed areas of colocalization, particularly along capillary walls in cases 2 and 3 (arrows) and areas of FHR5 alone in cases 1 and 2 (triangles). Notably, we did not detect areas of C3dg without FHR5 staining. (c) FHR5, C3b/iC3b/C3c, and C3dg glomerular locations correlate in C3G. We calculated the correlation of glomerular antigen locations in all available glomeruli from the three C3G cases (c). The Pearson’s correlation coefficient for all glomeruli from cases 1 (circles), 2 (squares), and 3 (triangles), and the median values of the correlations for each case (horizontal lines) are shown. The median values of the correlation coefficients (*r*) for each case were as follows: C3b/iC3b/C3c with FHR5: 0.73 (case 1), 0.76 (case 2), and 0.68 (case 3); FHR5 with C3dg: 0.71 (case 1), 0.90 (case 2), and 0.5 (case 3); and C3b/iC3b/C3c with FHR5: 0.68 (case 1), 0.81 (case 2), and 0.58 (case 3). MMF, mycophenolate mofetil. Original magnification ×400. Bars = 100 μm.

We next assessed the relationship between glomerular C3 and FHR5. Glomerular FHR5 staining intensity positively correlated with the glomerular staining intensities of C3b/iC3b/C3c, C3dg, and C5b9 (Figure 3a). Glomerular C3b/iC3b/C3c and C3dg positively correlated with glomerular C5b9. Glomerular C3b/iC3b/C3c and C3dg intensities also correlated significantly.

In 3 cases of C3GN, we performed double immunofluorescence staining for (i) FHR5 and either C3b/iC3b/C3c or C3dg, and (ii) C3b/iC3b/C3c and C3dg (Figure 3b). At the time of biopsy, serum C3 was normal in case 1 and low in cases 2 and 3. We noted areas of C3b/iC3b/C3c and C3dg codeposition (Figure 3b, right-hand column, arrows), and areas staining for either C3b/iC3b/C3c (Figure 3b, right-hand column, stars) or C3dg (Figure 3b, right-hand column, triangles) alone. Capillary wall (case 3) and mesangial FHR5 (cases 1 and 2) colocalized with C3b/iC3b/C3c (Figure 3b, left-hand column, arrows); however, there were also areas of glomerular C3b/iC3b/C3c staining without FHR5 (Figure 3b, left-hand column, cases 1 and 2, stars) and FHR5 without C3b/iC3b/C3c (Figure 3b, left-hand column, cases 2 and 3, triangles). Glomerular C3dg deposition was detected predominantly along capillary walls in cases 2 and 3, but was less marked and mesangial in location in case 1 (Figure 3b). Capillary wall FHR5 colocalized with C3dg (Figure 3b, middle column, cases 2 and 3, arrows). We also observed areas of FHR5 without C3dg deposition in cases 1 and 2 (Figure 3b, middle column, cases 1 and 2, triangles).

Antigen glomerular locations correlated positively in all glomeruli (Figure 3c). The median values of the antigen location correlations were 0.72 for C3b/iC3b/C3c with FHR5 (8 glomeruli from 3 cases; range 0.61 to 0.79), 0.75 for FHR5 with C3gd (5 glomeruli from 3 cases; range 0.5 to 0.91), and 0.74 for C3b/iC3b/C3c with C3dg (6 glomeruli from 3 cases; range 0.57 to 0.82).

### Glomerular FHR5 and C5b9 Staining Intensity Associates With Loss of eGFR in C3G

**Figure 4:**
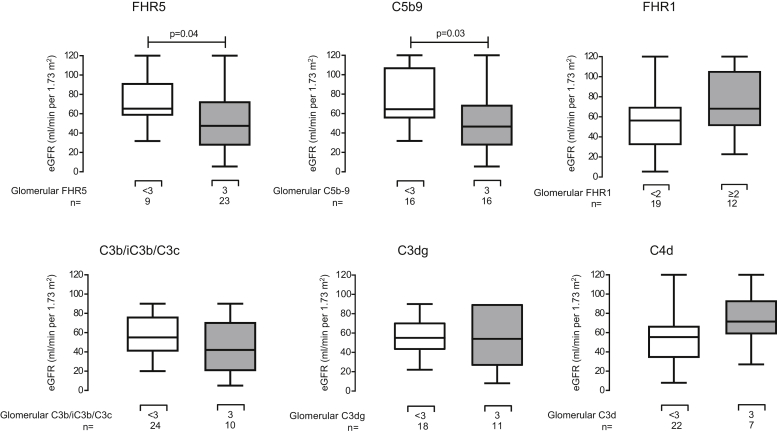
Glomerular factor H–related protein 1 (FHR5) and C5b9 staining intensity associated with lower estimated glomerular filtration rate (eGFR) at biopsy in C3 glomerulopathy. The eGFR at the time of biopsy was significantly lower in biopsies that had maximal staining intensities for FHR5 (*P* = 0.04, difference of medians 19.7 ml/min per 1.73 m^2^; 95% confidence interval [CI] 1.1–43.0) and C5b-9 (*P* = 0.03, difference of medians 14.86 ml/min per 1.73 m^2^; 95% CI 3.8–46.6). This was not seen for glomerular factor H–related protein 1 (FHR1), C3b/iC3b/C3c, C3dg, and C4d. *P* values are derived from Mann-Whitney *U* tests.

We next examined whether intense glomerular staining (grade 3+) associated with renal impairment (eGFR) at the time of biopsy (Figure 4). Both glomerular FHR5 and C5b9 negatively correlated with eGFR, whereas glomerular FHR1, C3b/iC3b/C3c, C3dg, and C4d did not. Our correlation excluded sclerosed glomeruli, so any relationships, such as that between glomerular FHR5 and eGFR at time of biopsy, were unlikely to be influenced by the prevalence of sclerosed glomeruli. Furthermore, we did not identify correlations between glomerular FHR5 intensity and the percentage of sclerosed glomeruli per biopsy (Pearson’s correlation coefficient *r* = 0.1993, *P* = 0.27). Glomerular FHR5 also correlated with an MPGN pattern at diagnostic biopsy (90% patients with vs. 33.3% without MPGN pattern, *P* = 0.049; odds ratio 18; 95% CI 1.6–201). Glomerular FHR5 deposition intensity did not correlate with age (*P* = 0.5), gender (*P* > 0.99), urine protein:creatinine ratio (*P* = 0.27), the presence of cellular crescents (*P* > 0.99), or endocapillary hypercellularity (*P* = 0.27) at the time of biopsy.

### Tubulo-interstitial FHR1 But Not FHR5 Was Detectable in C3G

**Figure 5:**
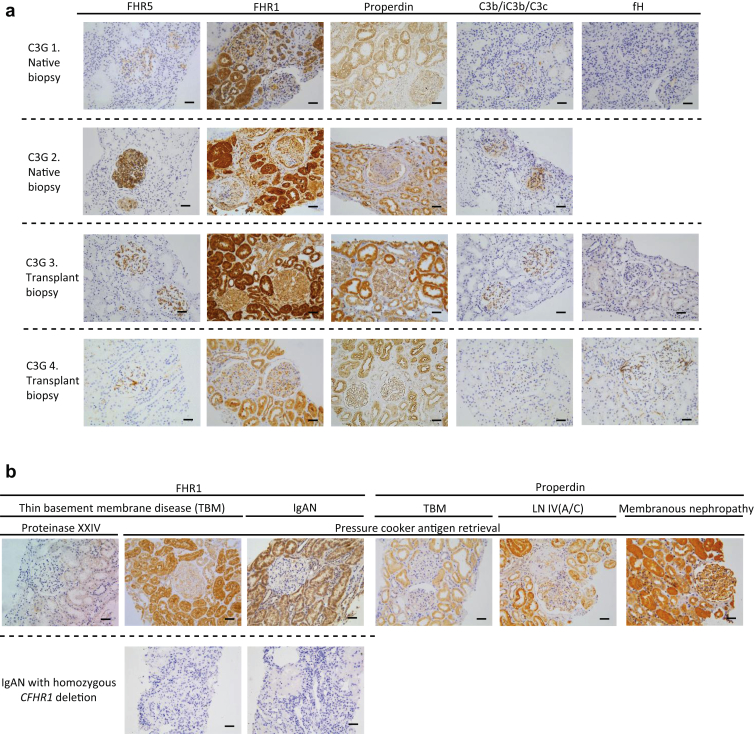
Tubulo-interstitial staining for FHR5 in C3 glomerulopathy. (a) Representative images of staining for factor H–related protein 5 (FHR5), factor H–related protein 1 (FHR1), factor H (fH), properdin, and C3b/iC3b/C3c. No tubulo-interstitial staining for FHR5 or fH was evident but there was strong tubulo-interstitial staining for both properdin and FHR1. (b) Tubulo-interstitial staining for properdin was also seen in other renal diseases (thin basement membrane disease [TBM], lupus nephritis [LN], and membranous nephropathy). Tubulo-interstitial staining for FHR1 was demonstrable in biopsies from patients with TBM and IgA nephropathy. Tubulo-interstitial FHR1 staining in TBM was still detectable but less intense when the proteinase XXIV enzyme was used instead of pressure cooker antigen retrieval. Glomerular and tubulo-interstitial FHR1 was absent in renal tissue from a patient with IgA nephropathy and FHR1 deficiency. C3G, C3 glomerulopathy; LN IV(A/C), lupus nephritis class 4, active and chronic. Original magnification ×200. Bars = 100 μm.

We did not detect significant FHR5 staining in the tubulointerstitium of either native or transplant C3G (Figure 5a); however, there was tubular cell staining for both FHR1 and properdin (Figure 5a). This staining did not correlate with proteinuria, eGFR, tubular atrophy, loss of 50% eGFR (data not shown), or tubular deposition of other complement proteins (Figure 5a). Tubular FHR1 staining was detected in non-C3G cases and was absent in a section from a patient with IgA nephropathy and FHR1 deficiency (Figure 5b). Notably, when we used pressure cooker heating for antigen retrieval, we were able to detect tubulo-interstitial FHR5 staining in different glomerulopathies, including C3G (Supplementary Figure 1). However, unlike the glomerular FHR5 staining evident using enzyme antigen retrieval, tubulo-interstitial staining could not be inhibited by pre-incubation of the primary antibody with purified FHR5, suggesting it was nonspecific.

## Discussion

Glomerular FHR5 was the most prevalent protein in native and transplant renal biopsies in our C3G cohort. FHR5 staining intensity associated with eGFR at the time of biopsy and correlated with the intensities of glomerular C3b/iC3b/C3c, C3dg, and C5b9 deposition. These findings support a role for FHR5 in complement-dependent glomerular injury, and FHR5 staining may improve C3G diagnosis. Murphy *et al.*^20^ demonstrated the presence of FHR5 in glomerular diseases, and we have demonstrated an association between IgA nephropathy severity and glomerular FHR5 deposition.^27^ It will be interesting to determine the prevalence of glomerular FHR5 deposition in a large population of patients with complement-dependent and complement-independent renal diseases. We identified an association between MPGN light microscopy pattern at diagnostic biopsy and a 50% loss of eGFR. Intense glomerular FHR5 deposition also associated with an MPGN pattern in our cohort. The interaction between glomerular FHR5 deposition and MPGN morphology requires further investigation. FHR5 and complement glomerular deposition would ideally be assessed in serial sections of the same glomeruli; however, the limited amount of surplus biopsy tissue precluded assessment of serial sections in most cases.

Glomerular FHR5 staining intensity correlated positively with the staining intensity of both C3b/iC3b/C3c and C3dg, which identify ongoing and previous complement activity, respectively. Our double-staining immunofluorescence protocols identified areas of FHR5 codeposition with C3b/iC3b/C3c and C3d. Therefore, FHR5 may bind both areas of ongoing (C3b/iC3b/C3c) and previous (C3dg) complement activity.

FHR5 and C5b9 were the only glomerular complement antigens to associate with renal impairment. The interplay of C3G activity, impaired glomerular filtration, and glomerular FHR5 and C5b9 deposition requires further investigation. Notably, glomerular C5b9 has been shown to persist in lupus nephritis following complement activity cessation,^34^ so may not be a reliable marker of ongoing glomerular C5 activation in C3G as well.

We made a number of interesting observations from transplant cases. Glomerular FHR5 deposition was identified in the absence of and predated detectable C3b/iC3b/C3c deposition and glomerular inflammation in one transplant patient. Whether this implicates FHR5 in early C3G pathogenicity or represents the sensitivity and affinity of FHR5 for otherwise undetectable C3 fragments requires further investigation. Specifically, a comparison of FHR5 deposition in protocol biopsies from patients with and without clinical signs of C3G recurrence is needed. Serial transplant biopsies during recurrent crescentic C3G showed FHR5 deposition correlated with initial disease activity, but was unaltered by C5 inhibition. This is consistent with a role for FHR5 in alternative but not terminal complement pathway activity. As evidenced by improved CD68-positive cell infiltration, clinical improvement following eculizumab treatment is likely secondary to reduced C5a production and inflammatory cell recruitment. Whether continued terminal pathway blockade would cause long-term disease improvement is unclear. Evidence from C3G animal models suggests alternative pathway activity in the absence of C5 reduces glomerular inflammation, but C3 glomerulopathy persists.^35^ The role of C5 inhibition in treating acute exacerbations of disease and its ability to modify long-term outcomes requires further study. Glomerular C4d was prevalent in both native and transplant C3G biopsies, suggesting the absence of C4d as a tool for diagnosing C3G is unreliable in our cohort, and the role of C4d in C3G requires further investigation.

In our protocols, glomerular FHR5 staining could identify complement activation in the absence of anti-C3b/iC3b/C3c staining. We speculate that, by comparing FHR5 and C3dg immunostaining patterns, it might be possible to identify areas within glomeruli where there is either ongoing (glomerular FHR5 without C3dg) or previous (glomerular FHR5 with C3dg) complement activation. Moreover, the combination of glomerular C5b9 and CD68 staining patterns could differentiate ongoing from previous glomerular C5 activation. This approach would rely on CD68 staining as a surrogate marker of complement C5a production. Consequently, ongoing C5 activation would be inferred if both C5b-9 and glomerular CD68 were positive; whereas previous C5 activation would be inferred if glomerular C5b-9 was positive in the absence of glomerular CD68.

Because FHR5 binds all immobilized C3 fragments *in vitro* (Supplementary Figure 2), the identification of glomerular C3b/iC3b/C3c deposits without FHR5 (Supplementary Figure 3) was unexpected. The structural conformation of C3c *in vivo* may hide the epitope that FHR5 binds *in vitro*. Alternatively, we speculate glomerular C3b/iC3b/C3c staining without FHR5 is C3c released from extrarenal complement activation that deposits in glomeruli but does not participate in complement activation^32^ and therefore does not interact with FHR5. In this scenario, comparing C3b/iC3b/C3c and FHR5 deposition could differentiate local from extra-glomerular complement activation.

Intratubular complement activation is associated with proteinuria and is properdin-dependent.^33^, ^36^, ^37^ We identified tubular FHR1 and properdin staining in our cases and these included those without proteinuria. The significance of FHR1 in this context is unclear. Tubulo-interstitial staining for FHR5 was absent when we used enzymatic antigen retrieval but became detectable when we used pressure cooker antigen retrieval. We considered this to be nonspecific staining, as we were unable to inhibit the staining with pre-incubation of the anti-FHR5 antibody with purified FHR5. Nevertheless, the role of the FHR proteins in the tubulointerstitium requires further investigation.

Mass spectrometry analysis of laser-captured glomeruli has identified alternative and terminal complement pathway components and FHR proteins in C3G.^38^ Using mass spectrometry spectra as semiquantitative evidence of glomerular protein abundance, Sethi *et al.*^38^ found C9, C3, and, specifically, C3dg were abundant in C3G, and DDD and C3GN cases had similar complement proteomic profiles. These observations seem consistent with our findings. By mass spectrometry, FHR1 was more prevalent and detected with higher mass spectrometry spectral counts than FHR5.^38^ In contrast, using immunohistochemistry, we found glomerular FHR5 to be more prevalent than FHR1; FHR5 was detected (at least 1+ intensity) in 96% (23 of 24) native and 100% (8 of 8) transplant biopsies; FHR1 was detected in 77% (17 of 22) native and 78% (7 of 9) transplant biopsies. Glomeruli FHR5 also appeared to be more abundant than FHR1; FHR5 was detected at 3+ staining in 71% (19 of 24) native and 63% (5 of 8) transplant biopsies, compared with FHR1 that we detected at 2+ intensity in 32% (7 of 22) native and 56% (5 of 9) transplant biopsies. Given the different research techniques and the experimental nature of protein quantification by spectra abundance, it is difficult to conclude why we did not replicate this finding in our cohort.

Our observational data cannot unravel the mechanisms that result in glomerular FHR5 deposition or its relationship with glomerular damage; however, we speculate that glomerular FHR5 interacting with C3 impairs the ability of fH to downregulate glomerular complement C3 activation. In addition, C3dg-bound FHR5 could facilitate C3 convertase formation and local C3 activation.^24^ FHR5 could have similar effects after binding directly to glomerular components, such as laminin-521 or -211.^39^ In addition, C3-bound FHR5 could impair complement receptor–dependent phagocytosis of deposited C3-coated immune complexes by mesangial cells and macrophages. In these scenarios, inhibition of glomerular FHR5 would be predicted to improve glomerular complement regulation and ameliorate complement-mediated glomerular inflammation and injury.

In summary, glomerular FHR5 deposition associates with C3G severity and colocalizes with C3 fragments *in vivo*. The prevalence of glomerular FHR5 binding to C3 fragments could be exploited to target therapeutic complement inhibitors to areas of complement activation in C3G.

## Disclosure

All the authors declared no competing interests.
