# Supplementary material for: Glomerular Complement Factor H–Related Protein 5 (FHR5) Is Highly Prevalent in C3 Glomerulopathy and Associated With Renal Impairment
Source: Kidney Int Rep. 2019 Jun 19;4(10):1387–400. doi: 10.1016/j.ekir.2019.06.008 (PMC6829196; doi:10.1016/j.ekir.2019.06.008)
Supplement: Supplementary File (PDF) [file mmc1.pdf]

Glomerular complement factor H related protein 5 (FHR5) is highly prevalent in C3 glomerulopathy and associated with renal impairment

Author list and affiliations:

Nicholas R. Medjeral-Thomas[1], Hilary Moffitt[1], Hannah J. Lomax-Browne[1], Nicholas Constantinou[1], Jack Galliford[2], Charles D. Pusey[1], Tom Cairns[2], H. Terence Cook[1], Matthew C. Pickering[1]

[1]Centre for Inflammatory Disease, Division of Immunology and Inflammation, Department of Medicine, Imperial College London, UK

[2]Renal and Transplant Centre, Imperial College Healthcare NHS Trust, UK.

Running title

FHR5 in C3 glomerulopathy.

Corresponding author:

Professor Matthew C. Pickering

Centre for Inflammatory Disease, Imperial College London, Hammersmith Campus, London W12 0NN

Email: matthew.pickering@imperial.ac.uk

Phone: +44 208 3832315

Fax: +44 208 3832379

Supplemental Figures

Medjeral-Thomas *et al*. Supplemental Figure 1

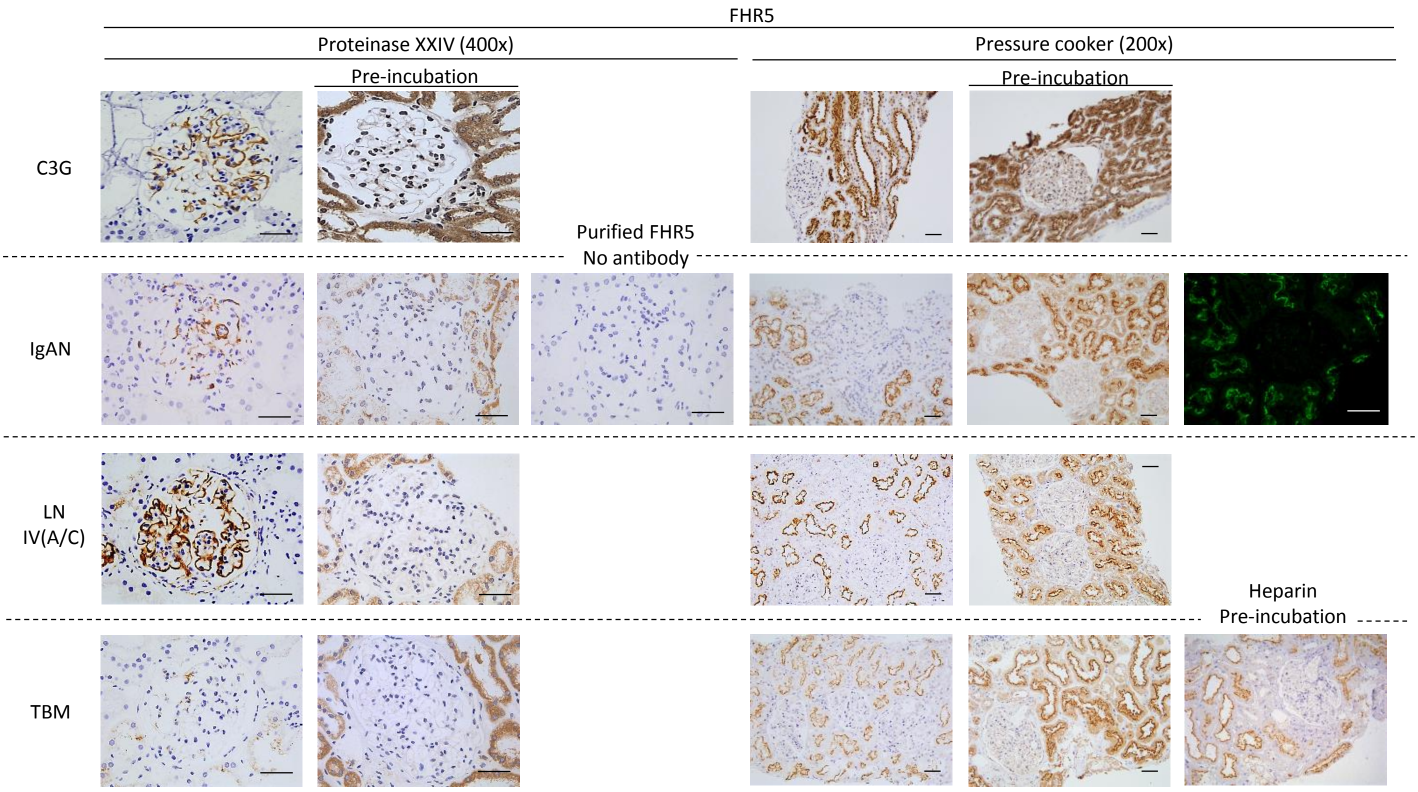

Supplemental Figure 1. Complement factor H related protein 5 (FHR5) immunohistochemistry (IHC) protocol optimisation

We optimised our IHC protocols to stain stored surplus formalin fixed and paraffin embedded renal biopsy tissue. When bacterial proteinase XXIV was used for antigen retrieval, glomerular FHR5 was detectable in C3 glomerulopathy (C3G) and other renal pathologies associated with glomerular complement deposition including IgA nephropathy (IgAN) and lupus nephritis (LN). Thin basement membrane (TBM) cases, that are not associated with complement activation and deposition, showed neither glomerular nor tubular staining. Glomerular FHR5 staining was eliminated when the primary antibody was pre-incubated with purified recombinant full length human FHR5 (R&D Systems #3845-F5) (column of images labelled 'Pre-incubation').

Glomerular FHR5 staining was dependent on the antigen retrieval method. Glomerular FHR5 staining was not detectable when pressure cooker heating was used instead of enzyme antigen retrieval. However, pressure cooker antigen retrieval revealed distinct tubular cell epithelial surface staining in all cases tested, including TBM. Tubular FHR5 staining after pressure cooker antigen retrieval did not associate with proteinuria and was not eliminated when the antibody was pre-incubated with purified human FHR5, which should eliminate antigen-antibody interactions (column of images labelled 'Pre-incubation'). Pre-incubation of a TBM section with heparin that would limit non-canonical glycosaminoglycan interactions also had no effect on tubular staining. The tubular staining was reproduced in a case of IgAN when we used a mouse monoclonal anti-human FHR5 antibody (Abnova #81494-B01P) with AF488-conjugated goat anti-mouse IgG (Thermofisher #A-11029). The staining could be explained by Fc interactions.

IgAN; IgA nephropathy. LN IV(A/C); lupus nephritis class IV (active/chronic). Images at 400x or 200x magnification. Bars represent 100 µm.

Medjeral-Thomas *et al*. Supplemental Figure 2

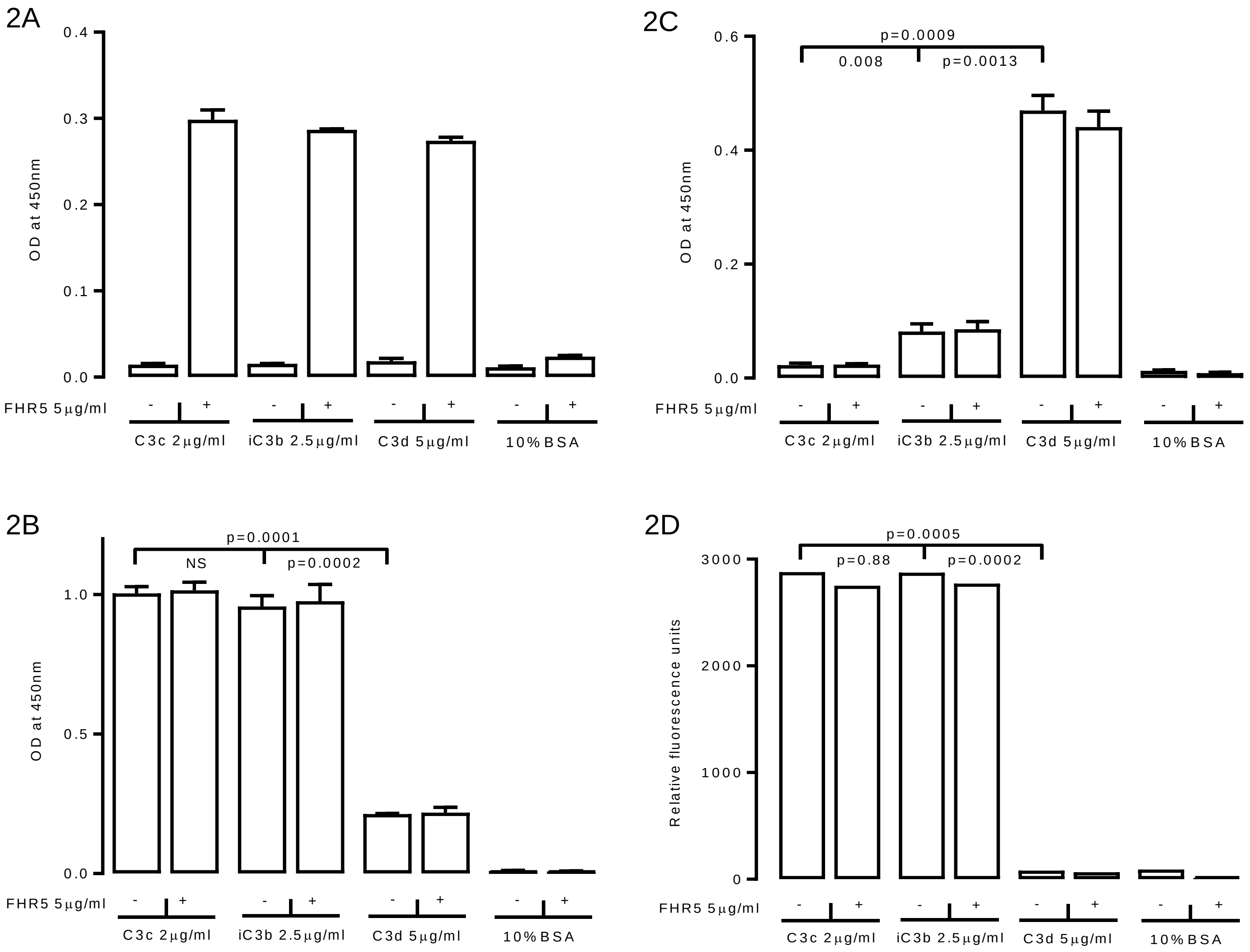

Supplemental Figure 2. Antibody specificity to purified human proteins by enzyme linked immunosorbent assay (ELISA)

Three ELISA plates were coated with purified human C3c (2µg/ml, Comptech #A116), iC3b (2.5µg/ml, Comptech #A115), C3d (5µg/ml, Comptech #A117) or 10% bovine serum albumin (BSA) overnight at 4°C. After washing, wells were incubated with either 5µg/ml purified human FHR5 (R&D Systems #3845-F5) or blocking buffer (1% BSA in 0.1% Tween PBS (1x)) for 45 minutes. After washing, wells were blocked for a further 45 minutes. We then added either (2A) rabbit anti-FHR5 (Abnova #81494-D01P), (2B) polyclonal rabbit anti-C3c (Dako #A0062), (2C) rabbit anti-C3d (Abcam #136916), or (2D) sheep anti-human C3c-FITC (ThermoFisher PA1-36179). For 2A-2C, bound antibody was detected using HRP-conjugated swine anti-rabbit IgG (Dako #P0260) and 3,3',5,5'-tetramthelybenzidine substrate (Dako #S1599). For 2D, bound antibody was detected by measuring relative fluorescence. Absorbance was calculated by subtracting the optical density (OD) at 540nm from 450nm.

(2A) The anti-FHR5 antibody could detect FHR5 incubated with surface bound complement (C)3c, iC3b and C3d by ELISA. We detected minimal binding of the anti-FHR5 antibody to the complement proteins in the absence of FHR5.

(2B) The anti-C3b/iC3b/C3c antibody used for immunohistochemistry (IHC) staining detected surface bound C3c and iC3b by ELISA with significantly greater sensitivity than it detected C3d or BSA. The antibody-antigen interactions were not affected by pre-incubation with FHR5.

(2C) The anti-C3d antibody used for IHC and immunofluorescence (IF) staining detected surface-bound C3d by ELISA with significantly greater sensitivity than it detected C3c, iC3b or BSA. The antibody-antigen interactions were not affected by pre-incubation with FHR5.

(2D) The anti-C3b/iC3b/C3c antibody used for IF staining detected surface bound C3c and iC3b by ELISA with significantly greater sensitivity than it detected C3d or BSA. The antibody-antigen interactions were not affected by pre-incubation with FHR5.

Medjeral-Thomas *et al*. Supplemental Figure 3

Immunofluorescence combination			Example	Consistent with antigen	Interpretation
**C3b/iC3b/C3c**	**C3dg**	**FHR5**			
+	+		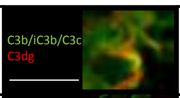	C3b/iC3b/C3c and C3dg	Ongoing and previous local C3 activation AND/OR C3c from local and/or systemic complement activation
+	-		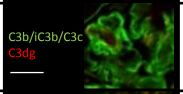	C3b/iC3b/C3c	Ongoing local C3 activation AND/OR C3c from local and/or systemic complement activation
-	+		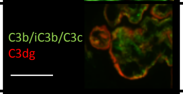	C3dg	Previous local activation
+		+	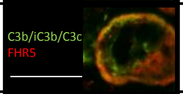	C3b/iC3b (or local C3c)	Ongoing local C3 activation
+		-	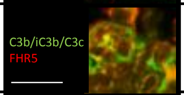	C3c	Systemic complement activation
-		+	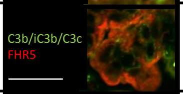	C3dg	Previous local activation
	+	+	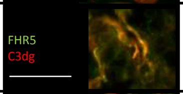	C3dg	Previous local activation
	-	+	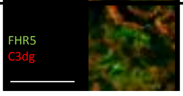	C3b/iC3b (or local C3c)	Ongoing local C3 activation

Supplemental Figure 3. The potential value of combination staining with antibodies to complement (C)3b/iC3b/C3c, C3d and factor H related protein 5 (FHR5)

Glomerular C3c is known to be cleared quickly in experimental nephritis so its detection in human glomeruli most likely indicates ongoing or recent complement activation. Conversely, glomerular C3dg is cleared slowly in experimental models so its presence in the absence of C3c in human glomeruli most likely indicates previous complement activation. We hypothesized that the combination of FHR5, C3b/iC3b/C3c and C3dg staining patterns could provide information on the nature of the glomerular complement C3 deposition: ongoing vs previous activation; and local (i.e. glomerular activation) vs systemic activation with subsequent glomerular deposition of complement fragments.

In this figure, we show magnified images of glomerular areas exemplifying each antigen combination and our interpretation of the staining.

Our data (supplemental figure 2) showed that FHR5 interacted with iC3b, C3c and C3d in vitro. Consistent with this all areas that stained with the anti-C3dg antibody also stained with the anti-FHR5 antibody. When these areas were not stained with the anti-C3b/iC3b/C3c antibody we concluded that they represented C3dg bound to FHR5. Areas of FHR5 staining without C3dg reactivity most likely represent FHR5 bound to C3b/iC3b. Notable, we did detect glomerular areas that stained with the anti-C3b/iC3b/C3c antibody but not with anti-FHR5 antibodies. We consider these to represent C3c that could derive from either complement activation in the circulation or released from complement activation within glomeruli. This interpretation depends on FHR5 interacting poorly with C3c in vivo.

Bars represent 50µm.
